# Evaluation of zinc and copper levels in vaginal tissues and whole blood: correlation with age

**DOI:** 10.1186/s12905-021-01215-6

**Published:** 2021-02-11

**Authors:** Anett Csikós, Bence Kozma, Edina Baranyai, Ida Miklós, Kindra Larson, Róbert Póka, Peter Takacs

**Affiliations:** 1 Molecular Biology Group, FemPharma, LLC, Debrecen, Hungary; 2 Department of Obstetrics and Gynecology, Faculty of Medicine, University of Debrecen, Debrecen, Hungary; 3 Department of Inorganic and Analytical Chemistry, Faculty of Science and Technology Agilent Atomic Spectroscopy Partner Laboratory, University of Debrecen, Debrecen, Hungary; 4 Department of Genetics and Applied Microbiology, Faculty of Science and Technology, University of Debrecen, Debrecen, Hungary; 5 Division of Female Pelvic Medicine and Reconstructive Surgery, Department of Obstetrics and Gynecology, Eastern Virginia Medical School, Norfolk, VA USA

**Keywords:** Ageing, Copper, ICP-OES, Vagina, Whole blood, Zinc

## Abstract

**Background:**

Zinc and copper are essential trace elements and play a crucial role in the homeostasis of connective tissues. In this study, we aimed to define zinc and copper levels in the vaginal tissue and establish whether a correlation exists between the zinc and copper levels either or both in whole blood or vaginal tissue samples and whether the finding correlates with the age of the patient or at least with her menopausal status.

**Methods:**

We collected whole blood and vaginal tissue samples from 32 women and measured their zinc and copper levels by inductively coupled plasma optical emission spectrometry. We have performed Student's *t* test to evaluate the differences in the mean levels of trace elements and multiple regression to evaluate the association between vaginal tissue zinc/copper levels and age, menopausal status, number of vaginal deliveries, and zinc/copper blood levels.

**Results:**

Zinc levels were significantly higher in both the vaginal tissues and whole blood samples than copper levels (*p* < 0.01). In the vaginal tissue samples, a strong positive correlation could be detected between zinc and copper levels (*r* = 0.82, *p* < 0.01). In the vaginal tissue, a negative correlation was found for zinc and copper levels with the age of women (*r* = − 0.27, *p* = 0.04 and *r* = − 0.56, *p* < 0.01). Multiple linear regression model (age, menopausal status, vaginal delivery and copper/zinc blood levels) showed that only age remained a significant predictor for zinc and copper vaginal tissues levels (*p* = 0.03, 95% CI − 2.28 to − 0.06; *p* = 0.004, 95% CI − 1.76 to − 0.34).

**Conclusions:**

Zinc and copper levels in the vaginal tissue decline with age. Out of the examined variables (age, menopausal status, vaginal delivery, and copper/zinc levels), only age is a significant predictor of vaginal zinc/copper levels.

## Background

Zinc (Zn) is an essential trace-metal of the human body, where 2–4 g [1] of it is distributed in the brain, muscle, bones, kidney, and liver, with the highest concentrations in the prostate and the eye [2]. The total amount of copper (Cu) in the human body is 75–100 mg, making it the third most common trace metal after iron and zinc [3]. Copper is needed for the iron metabolism and many other biological processes, like the neuropeptide synthesis and immune functions [4, 5], and vital for both antioxidant and pro-oxidant biochemical processes [6, 7].

The abnormality in the metabolism of trace elements in women is known to increase after menopause [8]. It is known that, generally, the metal intake decreases with age; therefore, it is not surprising that the zinc and copper level was reported to be low in older women [9]. In a population-based study of healthy elderly, a decrease in Zn, but an increase in Cu concentration was observed over 20 years of follow-up [10]. Another prior study reported low plasma zinc levels in French women older than 50 years, partly due to a decrease in dietary zinc intake [11]. Trace elements can affect the production and regulation of hormones, while female hormones affect mineral metabolism [12].

Animal experiments revealed that zinc affects the extracellular matrix of the vagina. Also, in aging mice and rats, the tissue concentration of zinc and copper decreases [13–15]. Histological and histochemical pictures of vaginas from mice with a zinc-deficient diet look similar to ovariectomized animals. Plasma zinc concentrations are significantly lower in buffalos with antepartum vaginal prolapse compared to healthy pregnant animals. In addition, the zinc level in the uterus is the lowest during menopause [16–18]. Zinc and copper are involved in many physiological processes. Vaginal tissues undergo significant remodeling during the lifetime of a woman. Compositions of the vaginal extracellular matrix (ECM) are changes during pregnancy, postpartum, and menopause [19].

We aimed to define zinc and copper levels in the vaginal tissue by inductively coupled plasma optical emission spectrometry (ICP-OES) method and establish whether a correlation exists between the zinc and copper levels either or both in whole blood or vaginal tissue samples and whether the finding correlates with the age of the patient or at least with her menopausal status. We have not found other studies investigating this question, though the changes in the trace metal levels can be important indicators (or causes) of disorders associated with aging. Our hypothesis was that vaginal tissue zinc, and copper levels decrease with aging.

## Methods

### Study design and selection of participants

Full-thickness vaginal wall biopsies and whole blood samples were collected from 32 women undergoing surgery for benign gynecological reasons at the University of Debrecen, Faculty of Medicine, Department of Obstetrics and Gynecology between 05/17/2017 and 04/20/2018 [20]. The study protocol complied with the Declaration of Helsinki principles and was approved by University of Debrecen, Faculty of Medicine, Department of Obstetrics and Gynecology, and all experimental methods and protocols were approved by the Hungarian National Institutional Review Medical Research Council (approval no. 7239-3/2017EIUG). All women signed written informed consent before participating in our research.

Exclusion criteria included women with osteoporosis, cancer, zinc deficiency, endometriosis, pregnancy, immunological and connective tissue diseases, recent use of vaginal or systemic hormone replacement therapy, and women with a prior pessary or intrauterine device (IUD) use. None of the participants indicated taking zinc or copper supplements. None of the patients used nicotine containing products or consumed excess amounts of alcohol. We prospectively collected relevant demographic and clinical data into a predefined database.

### Biological sample collection and processing

We have collected peripheral whole blood samples from the patients into 10 ml capacity BD Vacutainer® K2E (EDTA) plastic tube. We have divided each sample into five separate containers and stored them at − 20 °C for further analysis [20].

We have collected tissue specimens of vaginal wall biopsies in a standardized fashion from women undergoing abdominal or vaginal hysterectomy for benign gynecologic reasons [20, 21]. Tissues were transferred to polypropylene tubes and stored at − 70 °C for further analysis. Every participant received a unique study identification number after enrolment. We have transferred the coded samples to the laboratory and stored them for a maximum of 1 year. After thawing, the samples were processed immediately. During the laboratory measurements, the investigators were blinded to the persons providing the samples.

We have standardized the specimen collection because vaginal wall composition might vary throughout the vagina, and full-thickness biopsies of the anterior vaginal wall were cut from the midline area of the vault with Metzenbaum scissors as previously described [21]. We carefully avoided any crush injury to the site of the vaginal wall biopsy.

### Laboratory analysis

#### Sample pre-treatment

A validated analytical balance was used to weigh samples of vaginal tissues (Precisa ES225-SMDR, Precisa Gravimetrics AG, Switzerland). The samples were moved into glass beakers and placed into a drying cabinet to dry until constant weight. After measuring the dry mass of the samples, a pre-treatment of wet digestion was carried out before the elemental analysis. For the mineralization, we added 4 ml of 65% (m/m) nitric acid (Sigma-Aldrich, St. Louis, MO, USA) to the samples, and beakers were heated until complete dissolution. After cooling back to room temperature, an additional 1 ml of 30% (m/m) hydrogen peroxide (Sigma-Aldrich, St. Louis, MO, USA) was added. The resulted transparent solutions were transferred into volume calibrated plastic test tubes utilizing an ultrasound bath and diluted up to 10 ml with 0.1 M nitric acid prepared in ultrapure water (MilliQ, Millipore System, Merck, Germany) [20].

Whole blood samples were also treated with wet digestion at atmospheric pressure before the analysis: 1 ml of the blood was transferred into glass beakers and heated on an electric hot plate along with 5 ml concentrated nitric acid and 1 ml hydrogen peroxide. Before adding the hydrogen peroxide, 1 ml of ultrapure water was pipetted to the acid digested dry samples to avoid a too heavy a reaction. When the intense reaction generated by the peroxide stopped, the samples were poured into plastic tubes by continuously washing the beakers with 0.1 M nitric acid and diluted up to 10 ml final volume [20].

We have kept all samples in polypropylene tubes in which they were diluted and stored at 4 °C in a refrigerator until measurement.

#### Certified reference materials

Certified reference material of an artificial clinical control (Seronorm™, Sero AS, Billingstad, Norway) was used to validate the elemental analytical method. Trace metals were measured within the acceptance range given in the certification (relative standard deviation, RSD < 5%).

#### Elemental analysis

The elemental analysis of the pre-treated tissue and blood samples was carried out by inductively coupled plasma optical emission spectrometry (ICP-OES 5100, Agilent Technologies, Santa Clara, CA, USA). The measurements were conducted in SVDV (Synchronous Vertical Dual View) mode, gaining intensity data from the axial and radial view, simultaneously. An automatic sample introduction was applied (SPS 4, Agilent Technologies, Santa Clara, CA, USA), and the samples were measured in a randomized design [20]. We have performed measurements to generate a five-point calibration curve for the quantitative analysis of copper and zinc. We have diluted the calibration solutions from a multi-element standard of 1000 mg/l (ICP standard IV, Merck, Germany) with 0.1 M nitric acid in ultrapure water. We have expressed trace element concentration of vaginal tissues in milligrams per kilogram (mg/kg), and trace element concentration of whole blood samples in milligrams per liter (mg/l) [20].

### Statistical analysis

We have used SigmaStat (Systat Software, San Jose, CA, USA)/SPSS software (IBM, Armonk, NY, USA) for statistical analysis. We have performed Student's t-test to evaluate the differences in the mean levels of trace elements. The correlations between trace element concentrations in the blood / vaginal tissue samples and patients' age were assessed using the Pearson correlation coefficient. Multiple linear regression was calculated to predict vaginal tissue zinc and copper levels based on age, menopausal status, number of vagina deliveries and copper/zinc blood levels in which the vaginal tissue copper and zinc level was the dependent variable, the age, menopausal status, number of vagina deliveries and copper/zinc blood levels were independent variables. We have considered the differences significant if the *p* value was less than α = 0.05. Numerical results are presented as estimated mean ± standard deviation unless otherwise specified. Sample size calculation was performed with G*Power 3.1.9.7 Statistical Software for Windows (Heinrich Heine University, Düsseldorf, Germany). The following parameters were used: effect size of 0.3, the number of tested predictors of 1 and a total number of predictors of 4, and R^2^ of 0.4 (based on preliminary data). Sample size of 29 women is needed in order to achieve a power of 80% with a level of significance 5%.

## Results

Thirty-two women were enrolled in our study. The clinical and demographic characteristics of the participating women can be found in Table 1.

**Table 1:** Clinical and demographic characteristics of pre- and postmenopausal women

Features of the women	Results
Age (year, mean, SD)	62.5 ± 13.11
Premenopausal status, *n* (%)	7 (21.87%)
Premenopausal age (mean, SD)	44.4 ± 9.1
Postmenopausal status, *n* (%)	25 (78.13%)
Postmenopausal age (mean, SD)	67.6 ± 8.9
Number of pregnancies (mean, SD)	2.78 ± 1.26
Number of deliveries (mean, SD)	2.18 ± 1.14
BMI (body mass index) (mean, SD, kg/m^2^)	28.41 ± 4.78

We have analyzed the vaginal and whole blood zinc and copper concentrations and recorded the relevant clinical information (age, menopausal status, vaginal parity). Table 2 shows the zinc and copper levels, both in the vaginal tissue and whole blood samples of the patients.

**Table 2:** Zinc and copper concentration in the vaginal tissue and whole blood of women

	Zn mean (SD)	Cu mean (SD)	*p* value
Women (*n* = 32)			
Tissue (mg/kg)	57.86 (26.09)	15.89 (18.58)	< 0.01
Whole blood (mg/l)	4.84 (0.82)	0.84 (0.29)	< 0.01

Zinc levels were significantly higher in both the vaginal tissues [zinc vs. copper (mg/kg) 57.86 ± 26.09 vs. 15.89 ± 18.58, *p* < 0.01] and whole blood samples compared to copper levels [zinc vs. copper (mg/l) (4.84 ± 0.82 vs. 0.84 ± 0.29, *p* < 0.01].

**Fig. 1:**
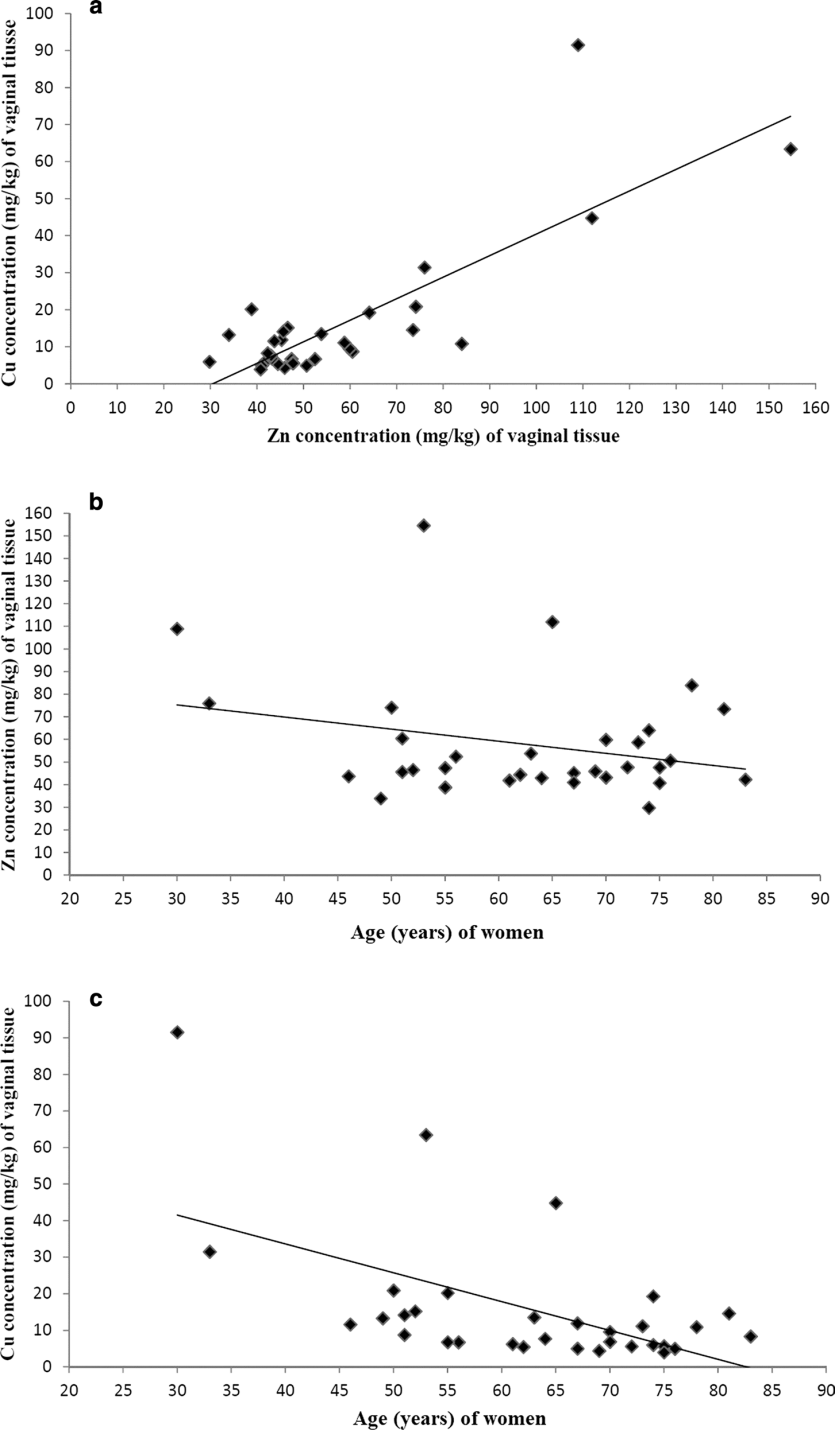
Correlation analysis of zinc and copper levels in the vagina. **a** Correlation between vaginal tissue zinc (Zn) and copper (Cu) levels (mg/kg). A significant strong positive correlation could be detected between zinc and copper vaginal tissue levels (*r* = 0.82, *p* < 0.01). **b** Correlation between vaginal tissue zinc (Zn) levels (mg/kg) and the age (years) of women. A significant negative correlation was found between zinc levels and the age of women (*r* = − 0.27, *p* = 0.04). **c** Correlation between vaginal tissue copper (Cu) levels (mg/kg) and the age (years) of women. A significant negative correlation was found between copper levels and the age of women (*r* = − 0.56, *p* < 0.01)

In the whole blood samples, we found no significant correlation between the zinc and copper levels (*r* = 0.25, *p* = 0.16). However, in the vaginal tissue samples, a strong positive relationship could be detected between them (*r* = 0.82, *p* < 0.01) (Fig. 1). In the whole blood samples, the zinc and copper levels or zinc/copper ratio did not correlate with the patient's age. However, in the vaginal tissue, a negative correlation was found for both zinc and copper levels with the age of the patients (*r* = − 0.27, *p* = 0.04 and *r* = − 0.56, *p* < 0.01, respectively) (Fig. 1). In addition, the copper/zinc ratio showed a negative significant correlation with the age of patients (*r* = − 0.669, *p* < 0.01).

Multiple linear regression was calculated to predict vaginal tissue zinc and copper levels based on age, menopausal status, number of vaginal deliveries, and blood zinc or copper levels. All examined variables (age, menopausal status, vaginal delivery, and copper/zinc blood levels) were included in the regression model. A significant regression equation was found (F(4, 27) = 4.4, *p* = 0.006), with an R^2^ of 0.4. The multiple linear regression model showed that only age remained a significant predictor for both zinc and copper vaginal tissues levels (*p* = 0.03, 95% CI − 2.28 to − 0.06; *p* = 0.004, 95% CI − 1.76 to − 0.34).

## Discussion

After a thorough review of the relevant scientific literature, we believe that this is the first report on the determination of zinc and copper levels in human vaginal tissue and the correlation between trace metals concentrations in the whole blood and the vaginal tissue. In addition, we are the first to report a decline of vaginal tissue trace element levels (zinc and copper) with aging. We have found a significantly higher zinc tissue levels in the vagina compared to copper. This finding is consistent with other organ’s tissue levels where zinc levels are higher than copper [22]. The biological importance of much higher zinc levels in tissues than copper is not fully explained, but zinc is likely to participate in more molecular processes than copper.

We have found a strong significant positive relationship between zinc and copper vaginal tissue levels and revealed a significant correlation between the patient's age and the metal levels in the vaginal tissue. We have identified a weak negative correlation between the age and the zinc levels and a moderate negative relationship between age and copper levels in the vaginal tissue.

Tissue levels of zinc and copper decrease with aging in animals [13–15], but previously no data has been available regarding animal or human vaginal tissues. However, in the human uterus (corpus) during menopause, copper levels were seen to rise as zinc levels fell [23]. Similarly to our findings, Griesmann et al. found a negative relationship between copper and age in tissue samples from the liver [24]. Yücel et al. evaluated serum copper, zinc levels in breast cancer patients and healthy controls and they did not find any difference in copper, zinc levels in patients with breast cancer who were below or above age 50 [25]. Some studies revealed that the serum copper/zinc ratio was also useful index in indicating some malignancies next to the serum copper and zinc levels. Earlier studies found that in cancer cases, serum copper levels and Cu/Zn ratios were significantly increased and zinc concentrations were decreased or unchanged [25, 26]. Yücel et al. have found that the Cu/Zn ratio was significantly high in premenopausal women [25]. In this study in the whole blood samples the zinc/copper ratio did not correlate with the patient's age. In addition, the vaginal tissue copper/zinc ratio showed a negative significant correlation with the age of patients.

In our cohort, both zinc and copper vaginal tissue levels decreased with age, and neither zinc nor copper blood levels were a predictor of vaginal tissue levels, suggesting a complex regulatory mechanism for vaginal tissue zinc and copper levels.

Honore et al. found that in the low estrogen status of the postmenopausal uterus, the highest copper levels occurred with the lowest zinc levels and varied based on the sample collection sites on the uterus. Therefore, the effects of hormonal status appear to be different not only for copper and zinc but also to vary with the site of tissue sampling in the uterus [23]. However, in our multiple regression analysis, age was the only significant predictor, and menopausal status was not. In addition, we have standardized our tissue collection to minimize the effects on our results [21].

What role does age play in trace element shortage? Improper nutrition, which is especially noticeable in postmenopausal women, can result in low levels of trace elements and vitamin deficiencies. Some studies based on national surveys reported that daily intakes of zinc and copper in the elderly are frequently lower than recommended [27, 28]. Also, in a population-based study of healthy elderly, a decrease in zinc but an increase in copper blood concentration was observed over 20 years of follow-up [10]. In addition, a recent publication revealed that zinc-containing vaginal gel significantly improved vaginal atrophy related symptoms in postmenopausal women, suggesting that zinc replenishment may help to restore vaginal tissue quality [29].

When interpreting our findings, some constraints need to be considered. The small number of patients enrolled was the most important limitation of our study. Specimen collection from young and healthy premenopausal women is quite a challenge because these patients are much less likely to undergo a hysterectomy. In addition, we have measured the zinc and copper levels in the whole blood, which could be considered a weakness as well as one of the strengths since whole blood could be a better reflection on whole blood zinc status but not necessary for copper. It adds some value to our study that we are the first to define and analyze the zinc/copper levels in the human vaginal tissue and describe the effect of age on these trace elements vaginal tissue levels. Based on our findings, we recommend future research studies to adjust tissue levels of trace elements for age.

## Conclusions

In conclusion, the whole blood samples analysis showed no correlation between zinc and copper levels, but we identified a strong positive correlation between zinc and copper levels in the vaginal tissue. We also found a negative correlation between the age of women and both zinc and copper vaginal levels. Our results suggest that copper and zinc levels in the vaginal tissue decline with aging. Further studies may clarify whether these changes contribute to the development of conditions associated with aging or menopause.

## Data Availability

All data used and/or analyzed during the current study are available from the corresponding author on reasonable request.

## Abbreviations

BMI: Body mass index
Cu: Copper
ECM: Extracellular matrix
ICP-OES: Inductively coupled plasma optical emission spectrometry
IUD: Intra uterine device
RSD: Relative standard deviation
Zn: Zinc

## References

[1] Wapnir RA (1990). Protein nutrition and mineral absorption.

[2] Pfeiffer CC, Braverman ER (1982). Zinc, the brain and behavior. Biol Psych.

[3] Willis MS, Monaghan SA, Miller ML (2005). Zinc-induced copper deficiency: a report of three cases initially recognized on bone marrow examination. Am J Clin Pathol.

[4] Bonham M, O’Connor JM, Hannigan BM, Strain JJ (2002). The immune system as a physiological indicator of marginal copper status?. Br J Nutr.

[5] Uriu-Adams JY, Keen CL (2005). Copper, oxidative stress, and human health. Mol Asp Med.

[6] Osredkar J, Sustar N (2011). Copper and zinc, biological role and significance of copper/zinc imbalance. J Clin Toxicol.

[7] Harris ED (2001). Copper homeostasis: the role of cellular transporters. Nutr Rev.

[8] Favier A, Nève J, Chappuis P, Lamand M (1996). Relevance of trace element supplements in women of different ages. Therapeutic uses of trace elements.

[9] Hercberg S, Preziosi P, Galan P, Deheeger M, Papoz L, Dupin H (1991). Dietary intake of a representative sample of the population of Val-de-Marne; III. Mineral and vitamin intake. Rev Epidemiol Sante Publique.

[10] Baudry J, Kopp JF, Boeing H, Kipp AP, Schwerdtle T, Schulze MB (2019). Changes of trace element status during aging: results of the EPIC-Potsdam cohort study. Eur J Nutr.

[11] Favier A, Hercberg S, Arnaud J, Preziosi P, Galan P, Momcilovic B (1991). Zinc and copper intakes and status of French population (Val de Marne 1988 Survey). Trace Elements in Man and Animal 7.

[12] Margen S, King JC (1975). Effect of oral contraceptive agents on the metabolism of some trace minerals. Am J Clin Nutr.

[13] Lossow K, Kopp JF, Schwarz M (2020). Aging affects sex- and organ-specific trace element profiles in mice. Aging (Albany NY).

[14] Zhang B, Podolskiy DI, Mariotti M, Seravalli J, Gladyshev VN (2020). Systematic age-, organ-, and diet-associated ionome remodeling and the development of ionomic aging clocks. Aging Cell.

[15] Öztürk G, Akbulut KG, Afrasyap L (2008). Age-related changes in tissue and plasma zinc levels: modulation by exogenously administered melatonin. Exp Aging Res.

[16] Takacs P, Zhang Y, Candiotti K, Jaramillo S, Medina CA (2012). Effects of PPAR-delta agonist and zinc on vaginal smooth muscle cells collagen and tropoelastin production. Int Urogynecol J.

[17] Kelkar MA, Khar SK, Mandakhot VM (1989). Studies on antepartum prolapse of the vagina in buffalo-plasma trace element concentrations. Arch Exp Veterinarmed.

[18] Taneja SK, Kaur R (1990). Pathology of ovary, uterus, vagina, and gonadotrophs of female mice fed on Zn-deficient diet. Indian J Exp Biol.

[19] Farage M, Maibach H (2005). Lifetime changes in the vulva and vagina. Arch Gynecol Obstet.

[20] Csikós A, Takacs P, Miklós I (2020). Comparison of novel single nucleotide polymorphisms of zinc transporters with zinc concentration in the human blood and vaginal tissues. Biometals.

[21] Boreham MK, Wai CY, Miller RT, Schaffer JI, Word RA (2002). Morphometric analysis of smooth muscle in the anterior vaginal wall of women with pelvic organ prolapse. Am J Obstet Gynecol.

[22] Yaman M, Kaya G, Simsek M (2007). Comparison of trace element concentrations in cancerous and noncancerous human endometrial and ovary tissues. Int J Gynecol Cancer.

[23] Honoré LH, Salkie ML, Jajczay FL (1986). The influence of anatomical site and hormonal status on the copper and zinc levels of human uterine smooth muscle. Clin Biochem.

[24] Griesmann GE, Hartmann AC, Farris FF (2009). Concentrations and correlations for eight metals in human liver. Int J Environ Health Res.

[25] Yücel I, Arpaci F, Ozet A (1994). Serum copper and zinc levels and copper/zinc ratio in patients with breast cancer. Biol Trace Elem Res.

[26] Zowczak M, Iskra M, Torliński L, Cofta S (2001). Analysis of serum copper and zinc concentrations in cancer patients. Biol Trace Elem Res.

[27] Martínez Tomé MJ, Rodríguez A, Jiménez AM, Mariscal M, Murcia MA, García-Diz L (2011). Food habits and nutritional status of elderly people living in a Spanish Mediterranean city. Nutr Hosp.

[28] Pennington JA, Young BE (1991). Total diet study nutritional elements, 1982–1989. J Am Diet Assoc.

[29] Takacs P, Kozma B, Erdodi B, Jakab A, Larson K, Poka R (2019). Zinc-containing vaginal moisturizer gel improves postmenopausal vulvovaginal symptoms: a pilot study. J Menopausal Med.

